# The miR155HG/miR-185/ANXA2 loop contributes to glioblastoma growth and progression

**DOI:** 10.1186/s13046-019-1132-0

**Published:** 2019-03-21

**Authors:** Weining Wu, Tianfu Yu, Youzhi Wu, Wei Tian, Junxia Zhang, Yingyi Wang

**Affiliations:** 1 Department of Neurosurgery, The First Affiliated Hospital of Nanjing Medical University, Nanjing, Jiangsu 210029 China; 2 Department of Neurosurgery, Nanjing First Hospital of Nanjing Medical University, Nanjing, Jiangsu China

**Keywords:** GBM, miR155HG, ANXA2, P-STAT3, ceRNA

## Abstract

**Background:**

Glioblastoma multiforme (GBM) is the most common and aggressive form of astrocytoma among adult brain tumors. Multiple studies have shown that long non-coding RNAs (lncRNAs) play important roles in acting as molecular sponge for competing with microRNAs (miRNAs) to regulate downstream molecules in tumor progression. We previously reported that miR155 host gene (miR155HG), an lncRNA, and its derivative miR-155 promote epithelial-to-mesenchymal transition in glioma. However, the other biological functions and mechanisms of miR155HG sponging miRNAs have been unknown. Considering ANXA2 has been generally accepted as oncogene overexpressed in a vast of cancers correlated with tumorigenesis, which might be the target molecule of miR155HG sponging miRNA via bioinformatics analysis. We designed this study to explore the interaction of miR155HG and ANXA2 to reveal the malignancy of them in GBM development.

**Methods:**

The expression of miR155HG was analyzed in three independent databases and clinical GBM specimens. Bioinformatics analysis was performed to assess the potential tumor-related functions of miR155HG. The interaction of miR155HG and miR-185 and the inhibition of ANXA2 by miR-185 were analyzed by luciferase reporter experiments, and biological effects in GBM were explored by colony formation assays, EDU cell proliferation assays, flow cytometric analysis and intracranial GBM mouse model. Changes in protein expression were analyzed using western blot. We examined the regulatory mechanism of ANXA2 on miR155HG in GBM by gene expression profiling analysis, double immunofluorescence staining, chromatin immunoprecipitation and luciferase reporter assays.

**Results:**

We found that miR155HG was upregulated in GBM tissues and cell lines. Bioinformatic analyses of three GBM databases showed that miR155HG expression levels were closely associated with genes involved in cell proliferation and apoptosis. Knocking down miR155HG suppressed GBM cell proliferation in vitro, induced a G1/S-phase cell cycle arrest, and increased apoptosis. We also found that miR155HG functions as a competing endogenous RNA for miR-185. Moreover, miR-185 directly targets and inhibits ANXA2, which exhibits oncogenic functions in GBM. We also found that ANXA2 promoted miR155HG expression via STAT3 phosphorylation.

**Conclusion:**

Our results demonstrated that overexpressed miR155HG in GBM can sponge miR-185 to promote ANXA2 expression, and ANXA2 stimulates miR155HG level through phosphorylated STAT3 binding to the miR155HG promoter. We establish the miR155HG/miR185/ANXA2 loop as a mechanism that underlies the biological functions of miR155HG and ANXA2 in GBM and further suggest this loop may serve as a therapeutic target and/or prognostic biomarker for GBM.

**Electronic supplementary material:**

The online version of this article (10.1186/s13046-019-1132-0) contains supplementary material, which is available to authorized users.

## Background

Glioblastoma multiforme (GBM) is the most aggressive form of astrocytoma and is characterized by rapid progression and poor prognosis [1]. Studies have indicated that GBM development is associated with disrupted gene regulatory networks. In addition to aberrant gene expression in GBM, several reports have demonstrated a role for non-coding RNAs (ncRNAs), which lack protein coding capability due the lack of an open reading frame, in GBM progression [2]. Among these ncRNAs, long ncRNAs (lncRNAs) are defined as RNAs > 200 nucleotides in length and have been demonstrated to participate in diverse cellular processes including epigenetics and transcriptional and posttranscriptional regulation [3]. At the posttranscriptional level, lncRNAs can act as competing endogenous RNAs (ceRNAs) or molecular sponge that function by sponging microRNAs (miRNAs), short endogenous ncRNAs approximately 18–25 nucleotides in length, resulting in inhibiting the effects of miRNAs on target mRNAs [4].

The lncRNA miR155HG is transcribed from a gene located on chromosome 21q21 and consists of three exons that span 1.5 Kb. MiR155HG, also known as B-cell integration cluster, includes an imperfectly base-paired stem loop in exon 3 that is conserved across species and is a primary miRNA for miR-155 [5]. MiR155HG was initially thought to be involved in the human immune response. For example, Haasch et al. showed that transcriptional activation of miR155HG is an early and sustained T cell activation event [6]. Several oncological studies have shown that miR155HG is highly expressed in diffuse large and primary mediastinal B cell lymphomas [7]. MiR155HG can be induced by abnormal B-cell receptor in Hodgkin lymphoma [8], and miR-155 was also shown to be upregulated by mutant p53 and facilitate invasion of breast cancer cells [9]. Our group previously reported that the miR155HG/miR-155 axis exhibits an oncogenic function in glioma by promoting epithelial-to-mesenchymal transition [10]. However, other biological mechanisms of miR155HG, such as potential ceRNA functions, and the upstream regulation of miR155HG in astrocytoma have remained unknown.

Annexin A2 (ANXA2) is a calcium-dependent phospholipid-binding protein with demonstrated roles in stimulating fibrinolytic processes, degrading extracellular matrix, and promoting angiogenesis [11]. Recent reports have shown that ANXA2 is aberrantly expressed in a wide variety of tumors [12–15] and has been implicated in various processes of tumorigenesis, including cell invasion [16], proliferation [17] and neovascularization [18]. Previous studies reveal invasion function for ANXA2 in glioma and suggest its role as a potential diagnostic and prognostic marker for glioma [19, 20]. However, whether ANXA2 is involved in the oncogenic functions of miR155HG in glioma has not been determined.

Through bioinformatical analysis and experimental analyses, here we confirmed that miR155HG is overexpressed in GBM and acts as a ceRNA for the tumor suppressor miR-185 to upregulate ANXA2. We also showed that ANXA2 promotes GBM growth and miR155HG expression by activating STAT3. Our results demonstrate that miR155HG, miR-185 and ANXA2 form a signaling loop that promotes malignant phenotypes in GBM.

## Methods

### Public human astrocytoma databases, GBM specimens and cell lines

Three public human astrocytoma databases (TCGA, CGGA and Rembrandt) were described in our previous study [21]. A total of 24 GBM tissues and 15 pair-matched adjacent normal brain edematous tissues (collected postoperatively between April 2016 and February 2017) were collected from patients who underwent surgical removal of GBM tumors at the First Affiliated Hospital of Nanjing Medical University. Samples were frozen in liquid nitrogen immediately after isolation and stored at − 80 °C to avoid RNA deterioration. Tumor tissues were collected after participants signed written informed consent. The study protocol was approved by The Institutional Review Board of the First Affiliated Hospital of Nanjing Medical University. GBM diagnoses were confirmed by two independent pathologists. Patients recruited into this study received no preoperative treatments.

The normal human astrocyte cell line NHA and the human GBM cell lines U87, U251, Ln229, T98, and A172 were purchased from the Chinese Academy of Sciences Cell Bank (Shanghai, China). A primary GBM cell line GP1 was extracted in December 2016 from the tumor of a patient with a temporal GBM; a second primary GBM cell line GP2 was extracted in January 2017 from the tumor of a patient with a frontal GBM. All cell lines were stored in liquid nitrogen before use. Cell culture was performed as described previously [22].

### Quantitative real-time PCR (qRT-PCR) and western blotting

QRT-PCR and western blotting were performed as described previously [22]. The primer for miR155HG is F 5′-GAGTGCTGAAGGCTTGCTGT-3′, R 5′-TTGAACATCCCAGTGACCAG-3′, for β-actin is F 5′-TCACCCACACTGTGCCCA-TCTACGA-3′, R 5′- CAGCGGAACCGCTCATTGCCAATGG-3′. The antibodies for western blot analysis were: anti-ANXA2 (1:1000; Abcam, Cambridge, UK), anti-cell cycle-related proteins (cyclin E, cyclin D, CDK4, CDK6) (1:1000; Cell Signaling Technology, Danvers, MA, USA), anti-Bax (1:500; Santa Cruz Biotechnology, Dallas, TX, USA), anti-Bcl-2 (1:500; Santa Cruz Biotechnology), anti-caspase 3 (1:1000; Abcam) and anti-β-actin (1:1000, Cell Signaling Technology). Cells were treated with EGF Recombinant Human Protein Solution (Thermo Fisher Scientific, Waltham, MA, USA) or the SH-4-54 inhibitor of STAT3 phosphorylation (Selleck, Shanghai, China) according to the manufacturer’s protocol.

### Oligonucleotides, plasmid and transfection

To construct a plasmid expressing miR155HG, the full-length human miR155HG sequence or a mutated miR155HG sequence for miR-185-5p (NCBI Reference Sequence: NR_001458.3) was synthesized and inserted into the pCDNA3.1 vector to generate pCDNA3.1-miR155HG WT or MUT, respectively (Genechem, Shanghai, China). The hsa-miR-185-5p mimic, hsa-miR-185-5p inhibitor, and hsa-miR-scramble were chemically synthesized (Ribobio, Guangzhou, China). The sequence of ANXA2 and miR155HG siRNAs were as follows: si-miR155HG: sense, 5′-CUGGGAUGUU-CAACCUUAATT-3′, antisense, 5′-UUAAGGUUGAACAUCCCAGTT-3′; scramble: sense, 5′-UUCUCCGAACGUGUCACGUTT-3′, antisense, 5′-ACGUGACACGUU-CGGAGAATT-3′. ANXA2 siRNAs were chemically synthesized (Invitrogen, Shanghai, China). ANXA2 siRNA 1 target sequence is 5′-CUGGGAAGAAGGCUUC-CUUTT-3′, ANXA2 siRNA 2 target sequence is 5′-AAGGAAGCCUUCUUCCC-AGTT-3′, ANXA2 siRNA 3 target sequence is 5′- GGGAAGAAGGCUUCCU-UCATT-3′. Cells were transfected with oligonucleotides or plasmid using Lipofectamine 2000 Reagent (Invitrogen, Carlsbad, CA, USA) following the manufacturer’s instructions.

### Lentiviral packaging and stable cell lines

Lentiviruses carrying shRNA-miR155HG or shRNA-ANXA2 and the negative control lentivirus (sh-miR155HG sequence is 5′-CUGGGAUGUUCAACCUUAATT-3′; sh-ANXA2 sequence is 5′- CGGGATGCTTTGAACATTGAA -3′; sh-NC sequence is 5′-UUCUCCGAACGUGUCACGUTT-3′) were assembled in the human embryonic kidney cell line 293 T, and the viruses were collected according to the manufacturer’s manual (Genechem). Stably transfected cell lines were established by infecting U87 cells with lentiviruses using a lentiviral packaging kit purchased from Genechem, followed by puromycin selection.

### Chromatin immunoprecipitation (ChIP)

ChIP assays were performed as previously described [22]. The EZ-magna ChIP kit (Millipore, Bedford, MA, USA) was used according to the manufacturer’s protocol. Crosslinked chromatin was sonicated into DNA fragments in the range of 200–1000 bp and immunoprecipitated using rabbit anti-p-STAT3 antibodies (Abcam). Negative control samples were prepared using control rabbit anti-IgG antibody (Abcam), and rabbit anti-RNA Polymerase II antibody (Abcam) was used for positive control. After immunoprecipitation, the beads were washed sequentially with low-salt buffer, high-salt buffer, LiCl buffer, and TE buffer each for 5 min at 4 °C. The immunoprecipitated DNA was then eluted by incubation in 100 μl of elution buffer (0.1 M NaHCO_3_ and 1% SDS) containing 10 μg proteinase K (Millipore) at 62 °C for 2 h with rotation. The eluted DNA was purified using the columns and buffers in the kit and then re-dissolved in 50 μl of PCR-grade water. Immunoprecipitated chromatin was analyzed by qPCR using primers targeting the phosphorylated (p)-STAT3 (p-STAT3) binding regions in the human miR155HG promoter. The primer sequences used for ChIP-qPCR are binding region 1 (− 1982 to − 1972): F 5′-GAGACATCATTATTGTCATT-3′, R 5′-TAGGAGTCAAATACACCTG-3′; binding region 2 (− 1548 to − 1411): F 5′-ATGGGAAATTCAGAAAGGC-3′, R 5′-TGATCATATGAGGGAGGAGC-3′; and binding region 3 (− 275 to − 116): F 5′-TTAAGAACAAAGGTTGGAGC-3′, R 5′-TGTGACTCATAACCGACCAG-3′. PCR conditions were set according to the instructions provided in the SYBR Green Kit (Roche Applied Science, Upper Bavaria, Germany). Results were analyzed by agarose gel electrophoresis.

### RNA immunoprecipitation (RIP)

RIP assays were performed using U87 cell extracts with the EZ-Magna RIP RNA-Binding Protein Immunoprecipitation Kit (Millipore, Burlington, MA, USA) according to the manufacturer’s instructions. U87 cells were rinsed with cold PBS and fixed with 1% formaldehyde for 10 min. After centrifugation, cell pellets were collected and resuspended in NP-40 lysis buffer (Thermo Fisher Scientific, Waltham, MA, USA) supplemented with 1 mM PMSF, 1 mM DTT, 1% Protease Inhibitor Cocktail (Sigma-Aldrich, St. Louis, MO, USA) and 200 U/ml RNase Inhibitor (Life Technologies, Carlsbad, CA, USA). Lysates were subjected to high-speed centrifugation, and then 100 μl of the supernatant was incubated with RIP buffer containing magnetic beads conjugated with human anti-Ago2 antibody (Cell Signaling Technology). Mouse IgG (Cell Signaling Technology) was used as a negative control, while SNRNP70 (Cell Signaling Technology) was used as a positive control. Co-precipitated RNAs were detected by reverse transcription PCR. Total RNAs (input control), IgG and SNRNP70 were assayed simultaneously to evaluate the efficiency of Ago2-specific RNAs.

### Colony formation assays

Cells (4 × 10^2^) were seeded into cell culture dishes and cultured for 15 d. Cell colonies were fixed with 4% paraformaldehyde for 20 min and stained with 0.2% crystal violet. Images were captured and colonies (diameter > 0.5 mm) were counted using Image J software (National Institutes of Health, Arlington, VA, USA). All assays were repeated at least three times.

### EDU cell proliferation assays

EDU cell proliferation assays were conducted with the Molecular Probes EdU-Alexa imaging detection kit (Life Technologies). Cells treated for 48 h were incubated with 10 μM EdU for 2 h, fixed with 4% paraformaldehyde, permeabilized with 1% Triton X-100, and stained with the Alexa-Fluor 594 reaction cocktail for EdU and Hoechst 33342 (nuclei). Images were obtained using a fluorescence microscope (Olympus, Japan). All assays were repeated at least three times.

### Flow cytometric analysis

Transfected GBM cells in logarithmic growth were collected and processed with the Cell Cycle Staining Kit (Multi Sciences, Hangzhou, China) for cell cycle analysis. After washing with PBS, cells were fixed with 70% ice-cold ethanol, incubated with Cell Cycle Staining Kit for 30 min in the dark, and analyzed by flow cytometry. In other experiments, treated cells in logarithmic growth were harvested and stained with the Annexin V-FITC Apoptosis Detection Kit (Multi Sciences). After washing with PBS and incubating with Annexin V/propidium iodide for 30 min in the dark, cells were analyzed by flow cytometry.

### Immunohistochemistry (IHC)

Fresh intracranial tumor tissues from nude mice were fixed with 4% paraformaldehyde and then embedded in paraffin. Sections were incubated at 4 °C overnight with primary antibodies against ANXA2 (1:1000; Abcam) and p-STAT3 (1:500; Abcam). Sections were then incubated with secondary antibody (1:1000; Santa Cruz) for 2 h at room temperature and stained with diaminobenzidine until brown granules appeared.

### Fluorescence in situ hybridization (FISH)

RNA FISH was performed as described previously [22]. MiR155HG-Bio probe was synthesized from GoodBio (Wuhan, China); the sequence is 5′-CCTCCCACGGCAGCAATTTGTTCCA-3′. Frozen sections of fresh tissues were fixed with 4% formaldehyde for 10 min, washed with PBS, and then digested with Proteinase K for 5 min. After eliminating auto-fluorescence and blocking endogenous biotin, the sections were hybridized with probes overnight. Sections were then washed with pre-warmed 2× SSC at 37 °C for 10 min, 1 × SSC at 37 °C for 10 min, and 0.5 × SSC for 10 min. Tissue sections were then blocked with bovine serum albumin for 30 min at room temperature, followed by staining with 488-avidin (1:400) at room temperature for 50 min. Stained sections were washed with PBS for 5 min four times. Finally, tissue sections were mounted with a medium containing DAPI for 8 min in the dark and images were obtained with fluorescence microscope (Nikon, Japan).

### Dual-luciferase reporter assay

The ANXA2 3′-untranslated region (UTR) and the full miR155HG sequence containing miR-185-5p seed matching sites were amplified from human cDNA via PCR and cloned into the 3′ end of the pGL3-basic luciferase vector (Genechem). Mutated versions of each construct were generated by mutating the miR-185-5p seed site sequences (pGL3-wt or mut). The miR155HG promoter region sequence (2000 bp to 1000 bp upstream of transcription starting point) were also amplified and cloned into the 5′ end of the pGL3-basic luciferase vector. Mutated version was generated by deleting binding region sequences of p-STAT3 (wt or mut-pGL3). U87 cells seeded into 96-well plates were co-transfected with wt or mut report gene, the pRL-TK control (Promega, Madison, WI, USA) and miR-185-5p mimic or miRNA NC using Lipofectamine 2000 (Invitrogen). The wt or mut-pGL3 and the pRL-TK control were co-transfected into the cells, then treated with cell culture with or without SH-4-54 inhibitor of STAT3 phosphorylation. At 48 h after transfection, luciferase activity was determined using the Dual Luciferase Reporter Assay System (Promega, WI, USA) according to the manufacturer’s protocol. The relative luciferase activity was normalized to Renilla luciferase activity. All assays were performed in triplicate.

### Intracranial GBM mouse model

The animal experiments were approved by the Animal Management Rule of the Chinese Ministry of Health (document 55, 2001) and were performed conforming to the approved guidelines and experimental protocols of Nanjing Medical University. U87 cells (1 × 10^6^) stably expressing MCS-firefly luciferase for bioluminescence imaging were transfected with lentivirus expressing control shRNA, shRNA-ANXA2 or shRNA-miR155HG and then were intracranially injected into the frontal lobe of nude mice to generate GBM (*n* = 10 mice per group). Tumor volumes were measured by luciferase using a bioluminescence imaging system (Caliper IVIS Spectrum, PerkinElmer, Waltham, MA, USA) on days 1, 11, and 21 after implantation. The integrated flux of photons (photons/s) within each region was determined by the Living Images software package (Caliper Life Sciences). Mice were sacrificed when they were in deep coma. Brains were extracted, fixed in 10% formalin and then embedded in paraffin for IHC or frozen at − 80 °C for western blotting or FISH.

### Statistical analysis

Data are presented as the mean ± standard deviation (SD). Statistical analyses were performed using the Student *t* test to evaluate the significance of differences between groups, one-way ANOVA (Tukey’s post hoc) was used to determine the difference among at least three groups using SPSS v19.0 for Windows. (SPSS, IL, USA). Pearson’s correlations analysis and heat map microarray analysis were performed using Multiple Array Viewer 4.9 software (MEV). Kaplan–Meier survival analysis was performed using GraphPad 5.0 software. *P* < 0.05 indicates a significant difference.

## Results

### MiR155HG is overexpressed in GBM and miR155HG-related genes are enriched in cancer-associated processes

**Fig. 1:**
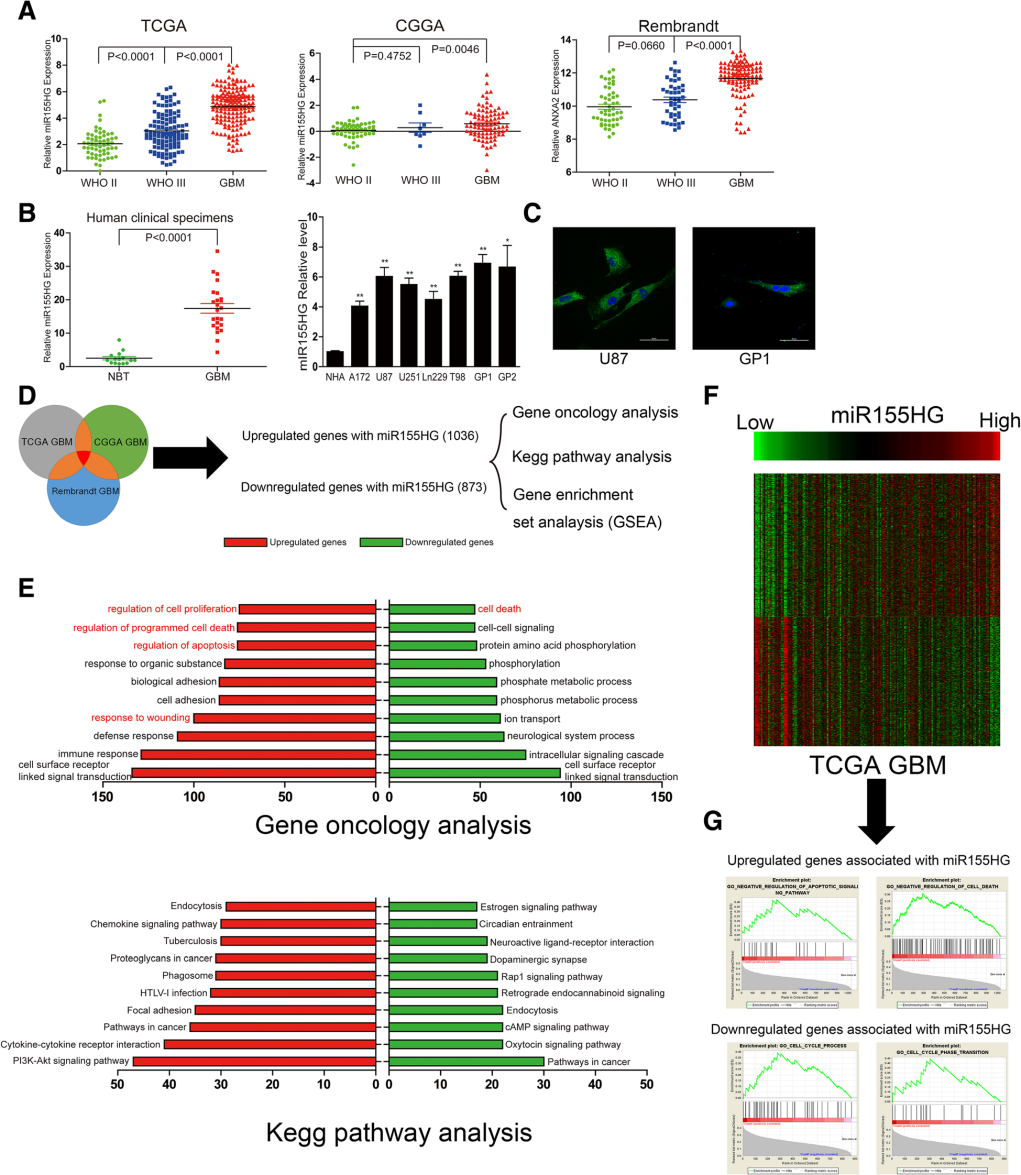
MiR155HG expression correlates positively with malignant degrees of glioma, and the miR155HG-associated genes were chiefly enriched in cancer related function. **a** Expression of miR155HG positively correlates with WHO grade in TCGA, CGGA and Rembrandt Public database. **b** Relative levels of miR155HG in the tumors and their adjacent normal brain tissues from 24 patients diagnosed as glioblastomas, and relative expressions of miR155HG in glioma cell lines NHA, A172, U87, U251, Ln229, T98, and primary glioma cells from two patients. **c** The distribution of miR155HG was evaluated via FISH in U87 and GP1 cells. **d-e** MiR155HG associated genes from overlapping CGGA, TCGA and Rembrandt databases were analyzed with KEEG pathway analysis, gene oncology analysis. **f-g** MiR155HG associated genes were analyzed with gene set enrichment analysis (GSEA) by TCGA genes data

To explore miR155HG expression in human astrocytoma tissues, we examined three public human astrocytoma databases (TCGA, CGGA and Rembrandt) and found overexpression of miR155HG in GBM (Fig. 1a). We also found that miR155HG expression was elevated in 24 GBM specimens compared with adjacent normal brain tissue from patients histologically diagnosed with GBM, and miR155HG was generally overexpressed in the GBM cells compared with NHA cells from normal brain tissue using fluorescent qPCR (Fig. 1b). FISH analysis revealed that miR155HG was primarily concentrated in the cytoplasm of GBM cells (Fig. 1c).

MEV software was used to perform a Pearson’s correlation analysis to identify genes associated with miR155HG expression in the TCGA, CGGA and Rembrandt GBM databases. The results identified 1037 up-regulated genes and 873 down-regulated genes from the overlap of the three databases, and these genes were examined using the DAVID Web tool (http://david.abcc.ncifcrf.gov/home.jsp) for Gene Oncology (GO) and KEGG Pathway enrichment analyses. GO analysis showed that the upregulated genes were primarily enriched in tumor progression processes, such as regulating apoptosis and cell proliferation. Moreover, KEGG pathway analysis indicated that the upregulated genes were closely associated with pathways activated in cancers, such as the PI3K-Akt signaling pathway, a prominent pathway in tumorigenesis and cancer progression (Fig. 1d-e). Furthermore, gene set enrichment analysis (GSEA) (http://www.broadinstitute.org/gsea/index.jsp) was used to examine genes that were expressed in TCGA GBM samples from patients with high miR155HG expression and those with low miR155HG expression. Genes expressed in patients with high miR155HG expression were primarily associated with reduced apoptosis and cell death, while genes with negative expression were primarily associated with cell cycle progression and cell cycle phase transition (Fig. 1f-g). Together these results suggested that miR155HG may be involved in the malignant phenotypes of GBM.

To evaluate the role of miR155HG in promoting malignant phenotypes of GBM, we generated an intracranial GBM mouse model by injecting U87 cells infected with lentivirus expressing control shRNA or shRNA-miR155HG. The tumor volumes in the group of mice treated with shRNA-miR155HG were smaller than those in the control at 11 and 21 days after implantation. Furthermore, mice treated with shRNA-miR155HG showed better survival than controls (Additional file 1: Figure S1A and B). These results indicated that miR155HG acted as oncogene in promoting GBM growth.

### MiR155HG sponges and downregulates miR-185-5p

**Fig. 2:**
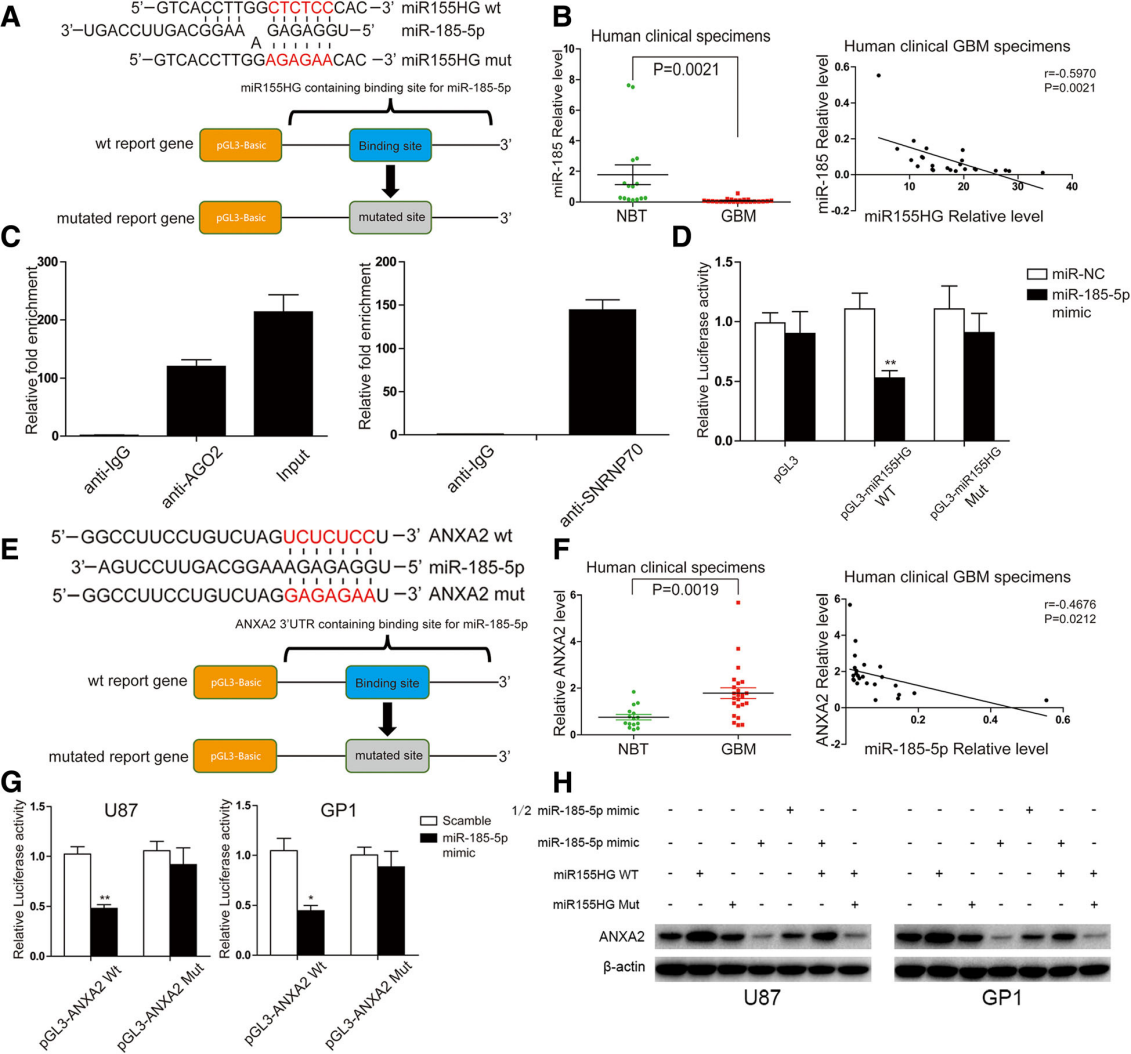
MiR155HG stimulated the ANXA2 expression by sponging endogenous miR-185-5p. **a** The binding site of MiR155HG and miR-185-5p was predicted by bioinformatics tools. **b** Expression levels of miR-185-5p in GBM tissues and adjacent normal brain tissues were analyzed by real-time PCR and normalized to U6. The correlation between MiR155HG and miR-185-5p in GBM tissues was applied with Pearson’s correlation coefficient (r = − 0.5970, *P* = 0.0021). **c** Luciferase assays was performed after transfection with miR-185-5p mimic and pGL3- miR155HG-wt or pGL3- miR155HG-mut into U87 cells as well as the internal control Renilla plasmid. Relative luciferase activity was analyzed after 48 h treatment. (*p < 0.05, **p < 0.01). **d** Amount of miR155HG bound to Ago2 or IgG measured by RT–qPCR after RIP assays with anti- IgG, anti-Ago2, anti-SNRNP70 and 10% input. **e** The binding site in 3’UTR of *ANXA2* mRNA for miR-185-5p was predicted by bioinformatics tools. **f** Expression levels of ANXA2 in GBM tissues and adjacent normal brain tissues were analyzed by western blot and normalized to β-actin. The correlation between miR-185-5p and ANXA2 in GBM tissues was applied with Pearson’s correlation coefficient (r = − 0.4676, *P* = 0.0212). **g** Luciferase assays was performed after transfection with miR-185-5p mimic and pGL3- ANXA2-wt or pGL3- ANXA2-mut into U87 and GP1 cells as well as the internal control Renilla plasmid. Relative luciferase activity was analyzed after 48 h treatment. (*p < 0.05, **p < 0.01). **h** The protein expression levels of ANXA2 were analyzed by Western blotting after 48 h transfection in U87 cells with pcDNA3.1, pcDNA3.1-miR155HG wt, pcDNA3.1-miR155HG mut, miR-NC or different amount of miR-185-5p mimic, respectively

Previous studies have shown that lncRNAs can act as a sponge for miRNAs, therefore we speculated that miR155HG may exhibit sponge activity. RNA hybrid bioinformatics tools showed that miR155HG contains a putative binding site for miR-185-5p as tumor suppressor in a wide range of tumors [23–26] (Fig. 2a). We found that miR-185-5p levels were lower in GBM tissues than in normal brain tissue, and that miR-185-5p was significantly negatively correlated with miR155HG in the same GBM samples (Fig. 2b; r = − 0.5970, *p* = 0.0021).

RNA-induced silencing complex (RISC) is an essential factor in the biological effect of miRNAs, and Ago2 is an elementary catalytic constituent of RISC that is involved in RNA cleavage [27]. To explore the possible interactions between miR155HG and miR-185-5p, RIP was performed in U87 cells. SNRNP70 protein, which interacts with U1 spliceosomal RNA [28], was used as a positive control. MiR155HG was predominantly enriched in beads containing anti-Ago2 antibody compared with those harboring control IgG (Fig. 2c). This result suggests that miR155HG is capable of sponging miRNA as ceRNA.

Then we constructed a luciferase reporter plasmid containing the putative miR-185-5p binding site from miR155HG, as well as a mutant construct in which the binding site was mutated (Fig. 2a). Co-transfecting miR-185-5p mimic decreased the relative luciferase activity in U87 cells transfected with wild-type plasmid, but had no impact on the mutant construct (Fig. 2d), which suggests that miR155HG directly binds miR-185-5p.

### ANXA2 is the target molecule of miR-185-5p

Previous studies demonstrated a potential role for ANXA2 in glioma [19]. Bioinformatics analytical tools TargetScan and miRNAWalk 2.0 showed that the 3′-UTR of *ANXA2* mRNA contained a seed sequence of miR-185-5p (Fig. 2e). To determine whether ANXA2 may be involved in the miR155HG-miR-185-5p axis in GBM, we first examined the expression levels of ANXA2 in frozen GBM tissue samples by western blot. We found that ANXA2 was highly expressed in GBM tissue but not in normal brain tissue (Fig. 2f and Additional file 1: Figure S1C). We next examined the correlation between miR-185-5p and ANXA2 in GBM tissue and found that miR-185-5p negatively correlated with ANXA2 (r = 0. − 4676, *P* = 0.0212; Fig. 2f). We generated luciferase constructs containing either the wild-type (WT) 3′-UTR of *ANXA2* mRNA or a mutated (MUT) sequence in which the miR-185-5p seed sequences were mutated. Luciferase assays showed that expression of miR-185-5p decreased the luciferase activity of the WT reporter but not the activity of the MUT reporter in U87 and GP1 cells (Fig. 2g).

We speculated that ANXA2 levels in GBM cells may be regulated by miR-185-5p and affected by its interaction with miR155HG. Indeed, transfection of a miR155HG expression vector increased ANXA2 levels in U87 and GP1 cells; however, the vector expressing miR155HG with mutated binding sites for miR-185-5p had no effect on ANXA2 levels. In addition, miR155HG-mediated elevation of ANXA2 was blocked by co-transfection with miR-185-5p mimic in a dose-dependent manner (Fig. 2h). Furthermore, inhibiting miR155HG by siRNA downregulated ANXA2 levels in U87 and GP1 cells, which could be reversed by treatment with miR-185-5p inhibitor (Additional file 1: Figure S1D). Together these results demonstrated that miR155HG may promote ANXA2 expression by modulating the capacity of miR-185-5p to bind the 3′-UTR of *ANXA2* mRNA.

### ANXA2 enhances the malignant phenotypes of GBM cells

As ANXA2 was the downstream molecule positively modulated by miR155HG via the ceRNA mechanism, we needed to investigate the function of ANXA2 to explain the oncological role of miR155HG in GBM. Bioinformatics analysis showed that ANXA2 expression was mostly expressed in GBM samples from TCGA, CGGA and Rembrandt database (Additional file 2: Figure S2A). GO analysis showed ANXA2 was closely associated with genes involved in cell apoptosis and proliferation (Additional file 2: Figure S2B).

**Fig. 3:**
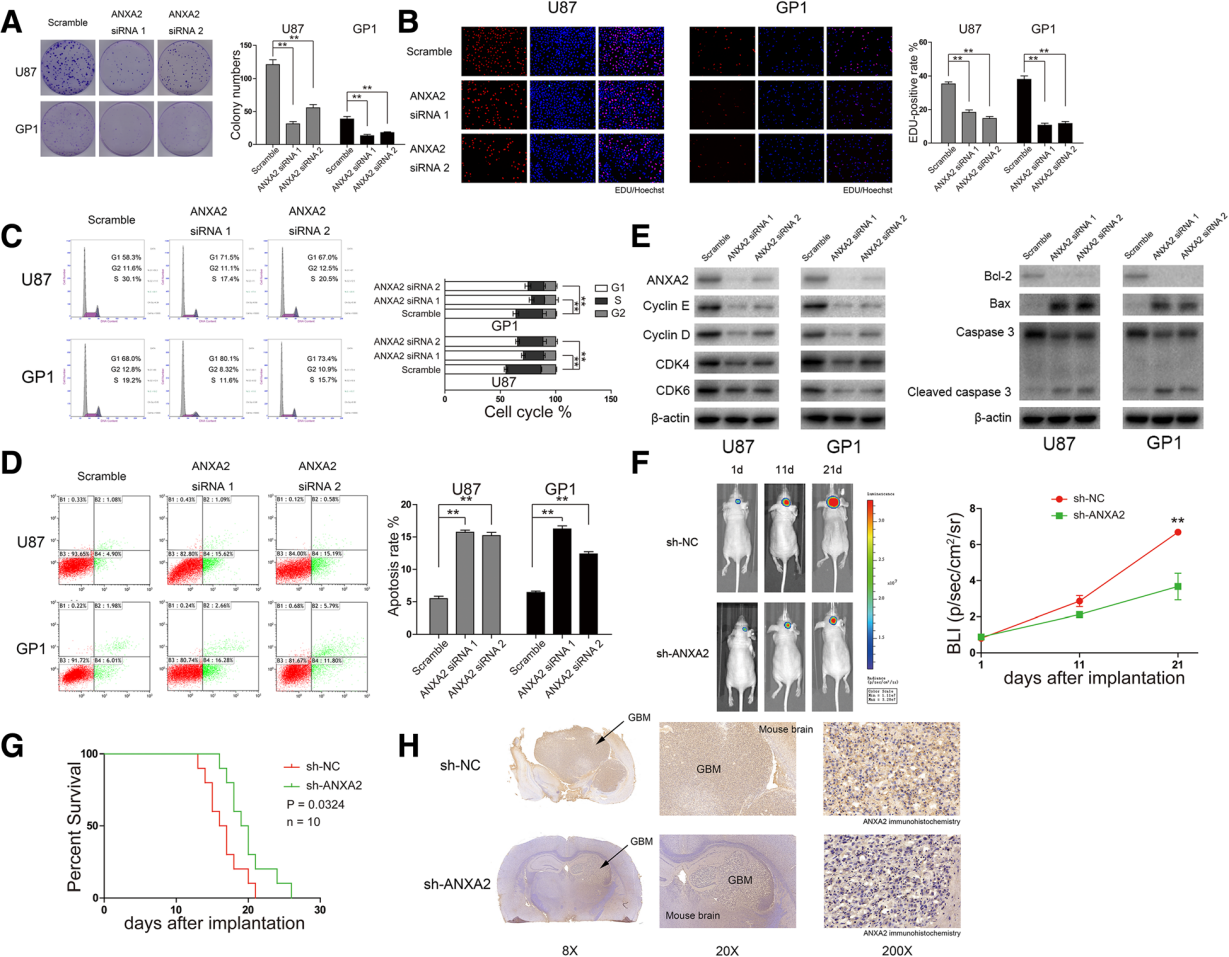
Downregulating ANXA2 expression inhibited GBM cells proliferation, cell cycle and apoptosis in vitro and *vivo*. **a** Colony formation assays in U87 and Primary glioblastoma cells (GP1) transfected with scramble, ANXA2 siRNA 1 and siRNA 2. Scale bar> 500 μm. **b** U87 and GP1 in EDU transfected with scramble, ANXA2 siRNA 1 and siRNA 2 after 48 h. Representative merged images were shown (original magnification, 200×). **c** The cell cycle was detected in U87 and GP1 transfected with scramble, ANXA2 siRNA 1 and siRNA 2 after 48 h. **d** The apoptotic cells were measured with flow cytometry in U87 and GP1 transfected with scramble, ANXA2 siRNA 1 and siRNA 2 after 48 h. **e** The protein related with cell proliferation, cell cycle and apoptosis was measured with immunoblotting in U87 and GP1 cells transfected with scramble, ANXA2 siRNA 1 and siRNA 2 after 48 h. All experiments above were performed 3 times, and average scores are indicated with error bars on the histogram.*P < 0.05, **P < 0.01. **f** U87 cells transfected with a lentivirus with sh-ANXA2 or sh-NC and a lentivirus containing luciferase were implanted in the brain of 10 nude mice, respectively. The tumor formation was assessed by bioluminescence imaging. The bioluminescent images were measured at days 1, 11 and 21 after implantation. **g** Overall survival was determined by Kaplan-Meier survival curves between sh-ANXA2 or sh-NC group, and a log-rank test was used to assess the statistical differences. **h** Two representative immunohistochemical image of tumors from groups of nude mice implanted with U87 cells, transfected with a lentivirus with sh-ANXA2 or sh-NC, were shown to compare the volume size of tumors

We next performed a series of experiments to evaluate the possible oncogenic function of ANXA2 in GBM. Colony formation and EDU assays were performed in cells transfected with ANXA2 siRNA to evaluate the effect of ANXA2 on proliferation. The effect of ANXA2 siRNA was shown in Additional file 3: Figure S3B. A significant reduction of proliferation was observed in U87 and GP1 cells transfected with ANXA2 siRNA compared with controls (Fig. 3a–b). Flow cytometry revealed that knocking down ANXA2 induced a G1/S arrest and decreased the percentage of cells in S phase (Fig. 3c). Flow cytometry also showed that knocking down ANXA2 remarkably increased apoptosis rates in GBM cells (Fig. 3d). Western blot results of proliferation- and apoptosis-associated proteins were consistent with the above results (Fig. 3e).

To examine the oncogenic ability of ANXA2 in GBM in vivo*,* nude mouse tumorigenicity assays were performed using a U87 xenograft model. U87 cells infected with fluorescent lentiviruses expressing sh-ANXA2 or controls were injected into nude mouse brains. The effect of ANXA2 knockdown in cells and GBM tissue was shown in Additional file 1: Figure S1E. In vivo imaging of the nude mice at 1, 11 and 21 d after implantation revealed that tumor growth was significantly inhibited at 21 d in the group with decreased ANXA2 expression (Fig. 3f). Mice injected with cells expressing sh-ANXA2 also showed better survival than control mice (Fig. 3g). IHC of tumor sections from the sh-ANXA2 and control groups confirmed decreased ANXA2 levels lead to inhibition of tumor growth in vivo (Fig. 3h). Taken together, our in vitro and in vivo results demonstrate that ANXA2 exhibits oncogenic functions in GBM cells and enhances the malignant phenotypes of tumors.

### MiR155HG and miR-185-5p participate in GBM growth by regulating the proliferation and apoptosis of GBM cells

**Fig. 4:**
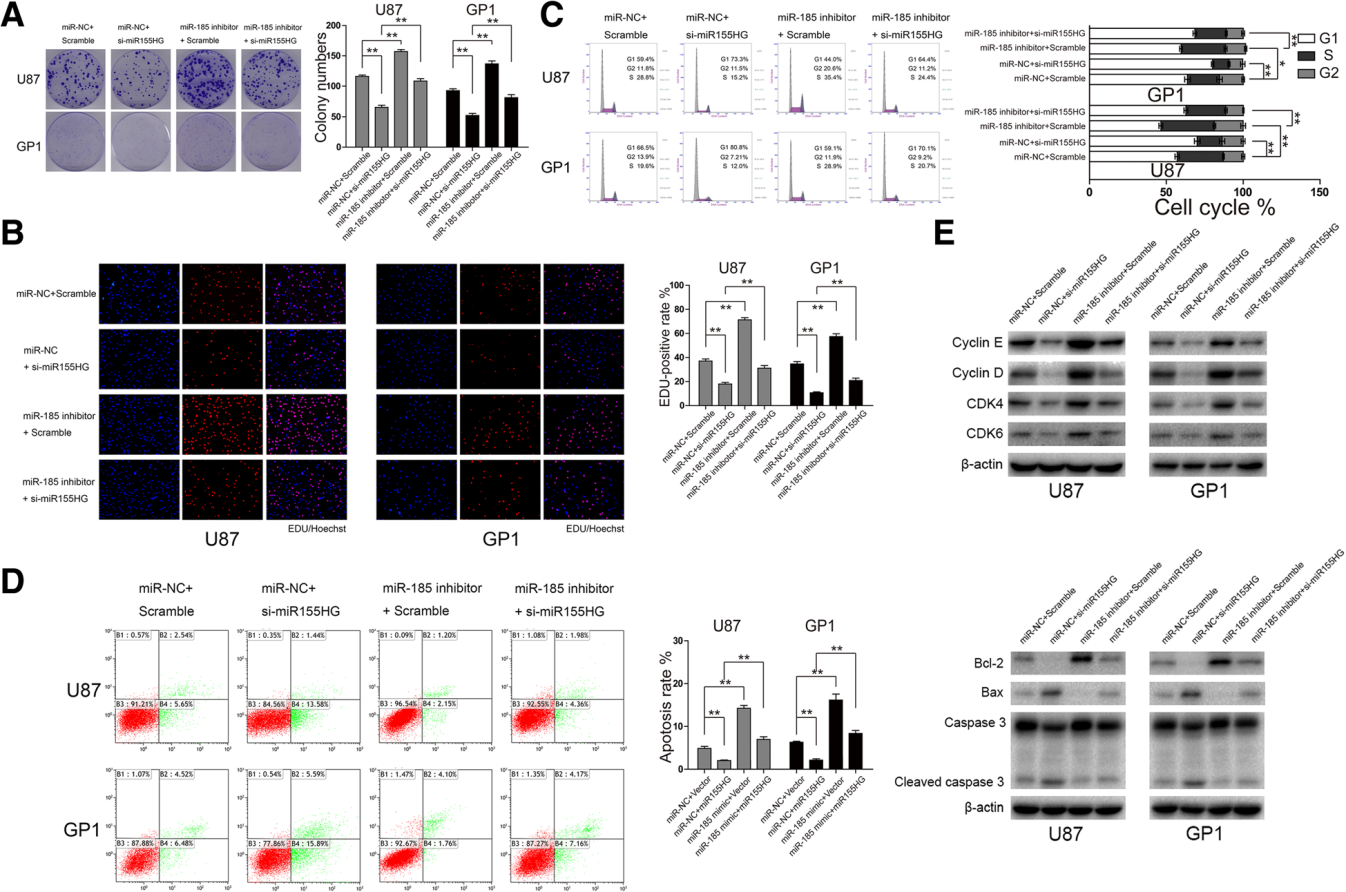
The impact of miR155HG and miR-185-5p on cell proliferation, cell cycle and apoptosis in GBM cells. **a** Colony formation assays in U87 and GP1 cells transfected with scramble, si-miR155HG, miR-185-5P inhibitor and si- miR155HG + miR-185-5P inhibitor. Scale bar> 500 μm. **b** U87 and Primary glioblastoma cells in EDU transfected with scramble, si-miR155HG, miR-185-5P inhibitor and si- miR155HG + miR-185-5P inhibitor after 48 h. Representative merged images were shown (original magnification, 200×). **c** The cell cycle was detected in U87 and Primary glioblastoma cells transfected with scramble, si-miR155HG, miR-185-5P inhibitor and si- miR155HG + miR-185-5P inhibitor after 48 h. **d** The apoptotic cells were measured with flow cytometry in U87 and Primary glioblastoma cells transfected with scramble, si-miR155HG, miR-185-5P inhibitor and si- miR155HG + miR-185-5P inhibitor after 48 h. **e** The protein related with cell proliferation, cell cycle and apoptosis was measured with immunoblotting in U87 and Primary glioblastoma cells transfected with scramble, si-miR155HG, miR-185-5P inhibitor and si- miR155HG + miR-185-5P inhibitor after 48 h. All experiments were performed 3 times, and average scores are indicated with error bars on the histogram.*P < 0.05, **P < 0.01

As ANXA2 plays a crucial tumor-promoting role in GBM and since ANXA2 levels are modulated by miR155HG and miR-185-5p, we hypothesized that miR155HG and miR-185-5p could interfere with the proliferation and apoptosis of GBM cells. Downregulating miR155HG levels in U87 and GP1 cells by siRNA reduced proliferation, blocked cell cycle progression, and stimulated apoptosis in GBM cells, and these effects were reversed by miR-185-5p inhibitor (Fig. 4a–d). Western blot analysis showed altered levels of proteins associated with proliferation and apoptosis, which was consistent with the above results (Fig. 4e). These results indicate that the biological role of miR155HG and miR-185-5p in GBM cells was due to regulating proteins associated with proliferation and apoptosis, and this may be partially by regulating ANXA2 expression.

### ANXA2 affected miR155HG expression in GBM cells via p-STAT3 levels

**Fig. 5:**
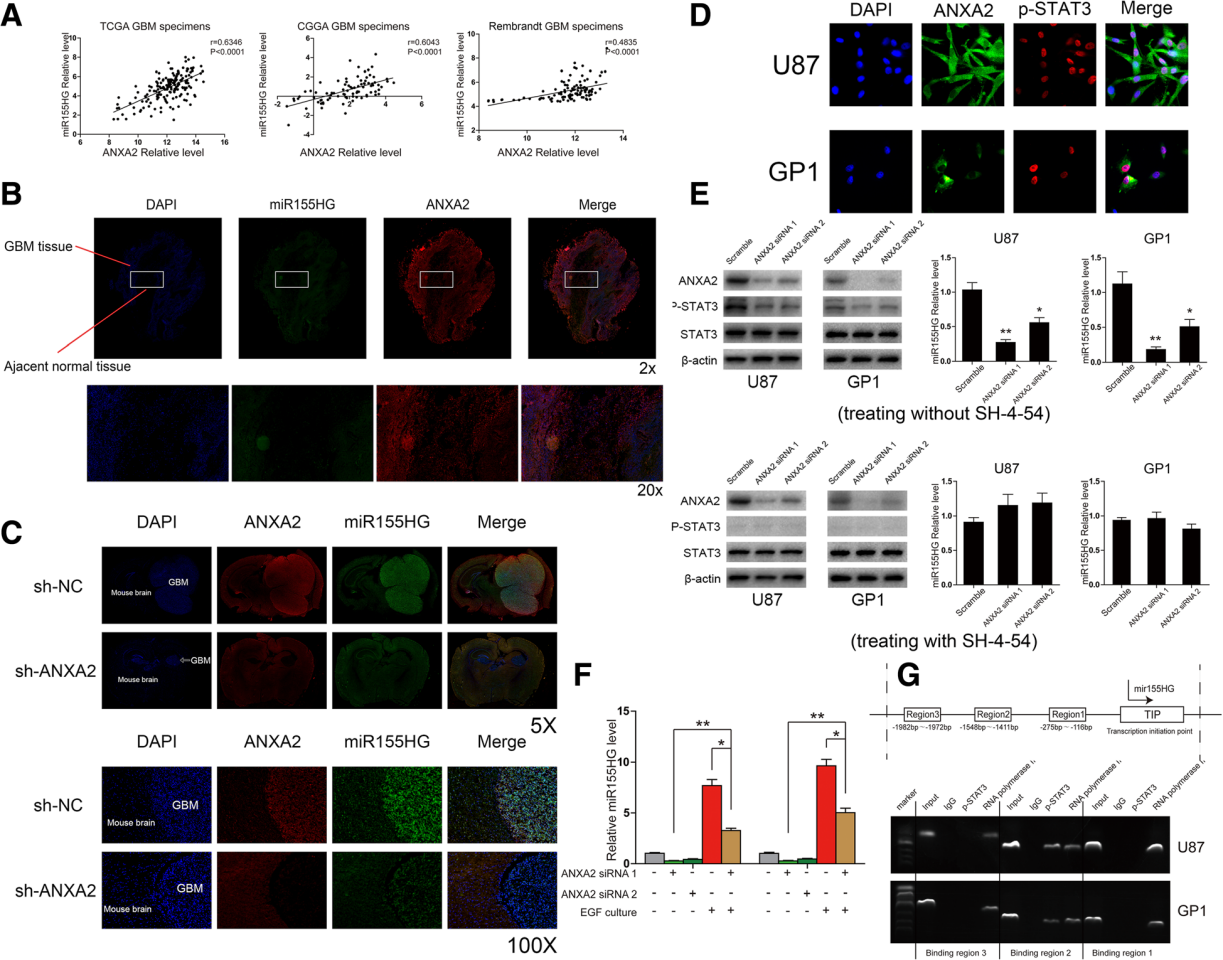
ANXA2 promote miR155HG expression via P-STAT3 in GBM cells **a** ANXA2 positively correlates with miR155HG in GBM specimens of three independent public database. **b** The level of ANXA2 and miR155HG was determined by immunofluorescence in boundary between invasive GBM and normal brain tissue from a GBM patient. **c** Two representative image of tumors from groups of nude mice implanted with U87 cells transfected with a lentivirus of sh-ANXA2 or sh-NC, were shown to compare the volume size of tumors and the expression of miR155HG via double immunofluorescent staining of the same frozen slice of tumor. **d** The specific localization of p-STAT3 and ANXA2 in U87 and GP1 cells was detected by immunofluorescence. **e** Q-PCR was performed to explore the level of miR155HG after downregulating ANXA2 in U87 and GP1 cells treated or untreated with inhibitor of STAT3 phosphorylation SH-4-54. **f** EGF-induced p-STAT3 rescued the ANXA2 knockdown-mediated downregulation of miR155HG in U87 and GP1 cells. **g** CHIP assays was performed to confirm the three putative binding region for p-STAT3 in promoter region of miR155HG

Our results show that miR155HG can interfere with ANXA2 expression by sponging miR-185-5p in GBM cells. Previous studies show that ANXA2 can act with AKT, STAT3 to promote downstream oncogenes [29, 30], we speculate if ANXA2 can contribute to miR155HG aberrantly overexpression in GBM cells in this way. Pearson’s correlation analysis showed that ANXA2 was significantly positively correlated with miR155HG in the TCGA, CGGA and Rembrandt GBM databases (Fig. 5a). We also found a positive correlation between ANXA2 and miR155HG in WHO II and WHO III astrocytoma patients in these independent public databases (Additional file 2: Figure S2C). Next, one representative GBM tissue exacted from a GBM patient during surgery was examined by FISH double staining and the results showed that ANXA2 and miR155HG were both significantly expressed in dense tumor tissues, but not in loose normal brain tissue (Fig. 5b). Then double-staining was performed to investigate to show that inhibiting ANXA2 caused a miR155HG downregulation in the brain of nude mice from Fig. 3g and interfered with tumor growth (Fig. 5c). We thus concluded that ANXA2 expression positively correlated with miR155HG levels in GBM.

Previous studies showed that ANXA2 modulates STAT3 phosphorylation (p-STAT3) levels to stimulate proliferation, angiogenesis, metastasis and epithelial to mesenchymal transition in breast cancer cells [30–32]. We found higher levels of p-STAT3 in GBM cells and tissues compared with normal brain cell and tissue(Additional file 3: Figure S3A), with distribution in nuclei in GBM cells (Fig. 5d). We thus wondered if ANXA2-mediated induction of miR155HG involved p-STAT3. We first found that decreasing ANXA2 resulted in reduced levels of p-STAT3 without impacting STAT3 levels in GBM cells (Additional file 3: Figure S3B). Inhibiting miR155HG also resulted in decreased ANXA2 and p-STAT3 in vivo (Additional file 3: Figure S3E). Knocking down ANXA2 resulted in reduced miR155HG expression, but this effect was not observed in GBM cells treated with a STAT3 phosphorylation inhibitor (Fig. 5e). Moreover, induction of STAT3 phosphorylation by Epidermal Growth Factor (EGF) [33, 34] could rescue the inhibitory effect of ANXA2 depletion on miR155HG expression (Fig. 5f and Additional file 3: Figure S3C). These results suggested that p-STAT3 might directly promote miR155HG expression in GBM.

Bioinformatics tools identified three putative binding regions for p-STAT3 in the miR155HG promoter. ChIP assays confirmed that p-STAT3 could bind putative binding region 2 (− 1548 bp to − 1411 bp) but not binding region 1 (− 275 bp to − 161 bp) and binding region 3 (− 1982 bp to − 1972 bp) in the miR155HG promoter (Fig. 5g). Two luciferase reporter plasmids containing either the entire miR155HG promoter region 2000 bp to 1000 bp upstream of the transcription start site (wt-pGL3) or the promoter deleted for binding region 2 (mut-pGL3) were transfected into GBM cells along with or without STAT3 phosphorylation inhibitor. While inhibiting STAT3 phosphorylation resulted in downregulation of luciferase activity driven by the wild-type promoter, the p-STAT3-driven luciferase activity on the mutated reporter remained unchanged (Additional file 3: Figure S3D).

Together these results indicated that the activated transcription factor p-STAT3 plays a key role in ANXA2-driven miR155HG expression and promotes GBM cell growth, through the DNA binding activity of the p-STAT3 transcription factor.

## Discussion

We previously revealed that miR-155, which is derived from miR155HG, functions in epithelial-mesenchymal transition in glioma [10]. In this report, we further showed that the lncRNA miR155HG is highly expressed in GBM, where it acts as a ceRNA to sponge miR-185-5p, thus promoting miR155HG downstream molecules level such as ANXA2. Our study clarifies the mechanism by which miR155HG positively regulates AXNA2 to sustain the malignant phenotypes of astrocytoma, particularly GBM.

Through bioinformatics analyses, we found that miR155HG was closely associated with the proliferative activity and apoptosis resistance of GBM in three independent GBM gene expression arrays. Knocking down miR155HG in GBM cells resulted in cell cycle arrest, decreased cell growth, and apoptosis. As lncRNAs have been proven to function as miRNA sponges [35], we hypothesized that miR155HG might also regulate gene expression by competing for shared miRNA response elements in GBM. Several studies have shown that miR-185 is involved in suppressing non-small cell lung cancer [36], gastric cancer [37], hepatocellular carcinoma [38] and prostate cancer [39] and is downregulated in glioma associated with inhibiting glioma cell invasion [40]. Another report showed that the lncRNA Linc00176 regulates the cell cycle by sponging miR-185 in hepatocellular carcinoma [41]. Here, we confirmed that lncRNA miR155HG binds to miR-185 to impact proliferation, cell cycle progression and apoptosis in GBM cell lines.

ANXA2, a 36-kDa protein that belongs to the family of calcium-dependent phospholipid binding proteins [11], is a DNA-binding protein that modulates DNA synthesis. Several studies showed that ANXA is involved in cell proliferation and cell cycle progression [42–45] in a variety of cancer cell types, such as breast cancer [18], hepatocellular carcinoma [46], colorectal cancer [47] and pancreatic cancer [48]. We found that ANXA2 was overexpressed in GBM. Based on TCGA, CGGA and Rembrandt GBM gene expression profiles, we found that ANXA2-associated genes were primarily enriched in cell proliferation and apoptosis. Knocking down ANXA2 inhibited proliferation and increased the G1/S cell cycle arrest apoptosis of U87 and GP1 cells. We also showed that ANXA2 is a key determining factor of survival that promotes the growth of intracranial GBM tumors in nude mice. We further found ANXA2 is a direct target of miR-185-5p, and its expression was perturbed by miR-185-5p. Taken together, these results established the miR155HG/miR-185-5p/ANXA2 axis, which underlies the biological mechanisms of miR155HG in GBM.

Silencing ANXA2 was previously reported to inhibit activation of STAT3 (p-STAT3) [31, 32, 49], and we confirmed that ANXA2 knockdown decreased p-STAT3 in GBM cells. Activated p-STAT3 forms homologous dimers and enters into the nucleus to function as a transcription factor to promote target gene expression [50]. Since constitutively activated STAT3 is closely associated with GBM [51, 52], we find that ANXA2-mediated elevated miR155HG levels is mainly due to p-STAT3 level. ChIP assay and luciferase reporter gene assay showed that p-STAT3 could bind to the miR155HG promoter region from − 1548 bp to − 1411 bp upstream of the transcription start site to stimulate miR155HG expression. This suggests that STAT3 phosphorylation is the critical role in driving lncRNAs regulation similar to miR155HG in malignant brain tumor.

Here we have established miR155HG/miR-185-5p/ANXA2 loop in GBM formation and progression. However, other interactions as epigenetic regulation between miR155HG and its downstream effector molecules remains obscure since lncRNA might recruit chromatin remodeling complex. More biological studies and clinical trials are needed to evaluate the practicality of targeting miR155HG for the treatment of GBM.

## Conclusions

**Fig. 6:**
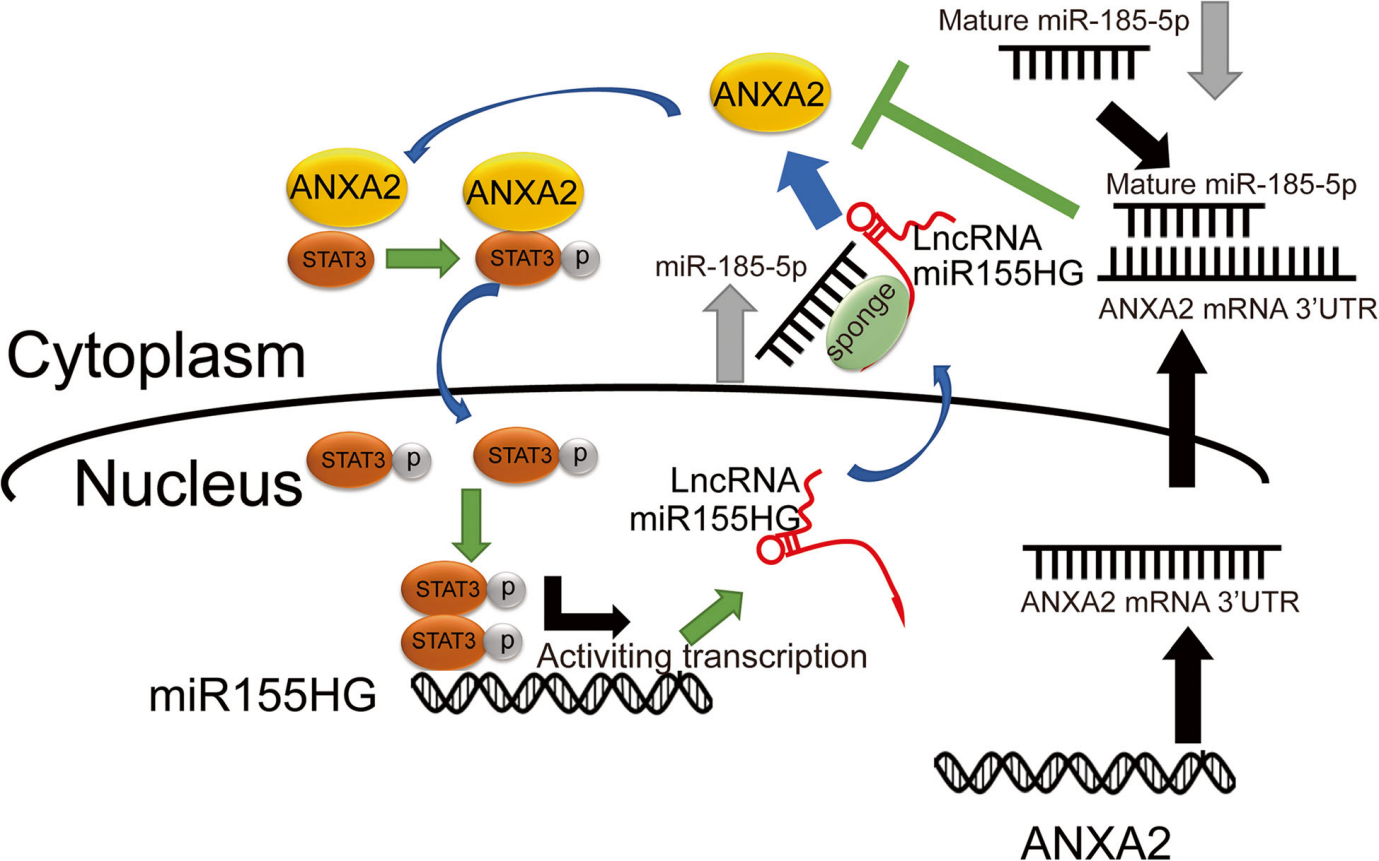
Schematic diagram of the relationship among miR155HG, miR-185-5p, p-STAT3 and ANXA2 in GBM. The binding of miR-185-5p to miR155HG leads to abolish miR-185-5p targeting ANXA2 3’UTR, which stimulated ANXA2-elevated level in cell. In turn ANXA2 then activates STAT3 phosphorylation, and STAT3 phosphorylation shifts from the cytosol to the nucleus, forms dimers and binds with promoter region to upregulate the expression of miR155HG

Our study suggested that the lncRNA miR155HG increases ANXA2 expression by sponging miR-185-5p to exert tumorigenic effects and that ANXA2 stimulates miR155HG level via ANXA2-driven p-STAT3 in GBM (Fig. 6). Thus, we have identified the miR155HG/miR-185-5p/ANXA2 loop and its mechanisms and biological effects in malignant brain tumors. This loop could serve as a novel therapeutic biomarker for GBM.

## Additional files

Additional file 1: **Figure S1.** (A) U87 cells pretreated with a lentivirus with sh-miR155HG or sh-NC and a lentivirus containing luciferase were implanted in the brains of nude mice, and tumor formation was assessed by bioluminescence imaging. The bioluminescent images were measured at days 1, 11 and 21 after implantation. (B) Overall survival from two groups of nude mice injected with U87 cells, transfected with sh-NC or sh-miR155HG lentivirus, was determined by Kaplan-Meier survival curves, and a log-rank test was used to assess the statistical significance of the differences. (C) Expression levels of ANXA2 in GBM tissues and adjacent normal brain tissues were analyzed by western blotting and normalized to β-catenin. (D) The protein expression levels of ANXA2 were analyzed by western blotting after 48 h transfection in U87 and GP1 cells with scramble, miR155HG siRNA 1, miR155HG siRNA 2, miR-NC or inhibitor, respectively. (E) The effect of sh-ANXA2 in U87 cell and tumor tissue of nude mice after implantation were analyzed by western blotting. (E) Downregulating ANXA2 contributed to the reduction of p-STAT3 level in GBM cells. (TIF 9482 kb)

Additional file 2: **Figure S2.** (A) Expression of ANXA2 in TCGA, CGGA and Rembrandt astrocytoma database. (B) ANXA2 associated genes from overlapping CGGA, TCGA and Rembrandt databases were analyzed with gene oncology analysis. (C) ANXA2 positively correlates with miR155HG in WHOII/III astrocytoma specimens of three independent public database. (TIF 3895 kb)

Additional file 3: **Figure S3.** (A) Expression levels of p-STAT3 in cell lines, GBM tissues and normal brain tissues were analyzed by western blot. (B) Downregulating ANXA2 contributed to the reduction of p-STAT3 level in GBM cells. (C) Overexpression STAT3 was constitutively activated by EGF in ANXA2-depleted GBM cells in U87 and GP1 cells. (D) Luciferase assays was performed after transfection with miR155HG promoter wt-pGL3 or miR155HG promoer mut-pGL3 as well as the internal control Renilla plasmid into U87 and GP1 cells. The cells then were treated with or without SH-4-54. Relative luciferase activity was analyzed after 48 h treatment. (*p < 0.05, **p < 0.01). (E) Two groups of representative immunohistochemical image of tumors from groups of nude mice implanted with U87 cells, transfected with a lentivirus with sh-miR155HG or sh-NC, were shown to compare the volume size of tumors and the expression of ANXA2 and p-STAT3 through serial slices of same section of tumor. (TIF 13681 kb)

## Abbreviations

EGF: Epidermal Growth Factor
CeRNAs: Competing endogenous RNAs
ChIP: Chromatin immunoprecipitation
FISH: Fluorescence in situ hybridization
GBM: Glioblastoma
GP1: Glioblastoma cell from patient 1
IHC: Immunoprecipitation
RIP: RNA immunoprecipitation
