# Supplementary figures and images for: The miR155HG/miR-185/ANXA2 loop contributes to glioblastoma growth and progression
Source: J Exp Clin Cancer Res. 2019 Mar 21;38:133. doi: 10.1186/s13046-019-1132-0 (PMC6427903; doi:10.1186/s13046-019-1132-0)

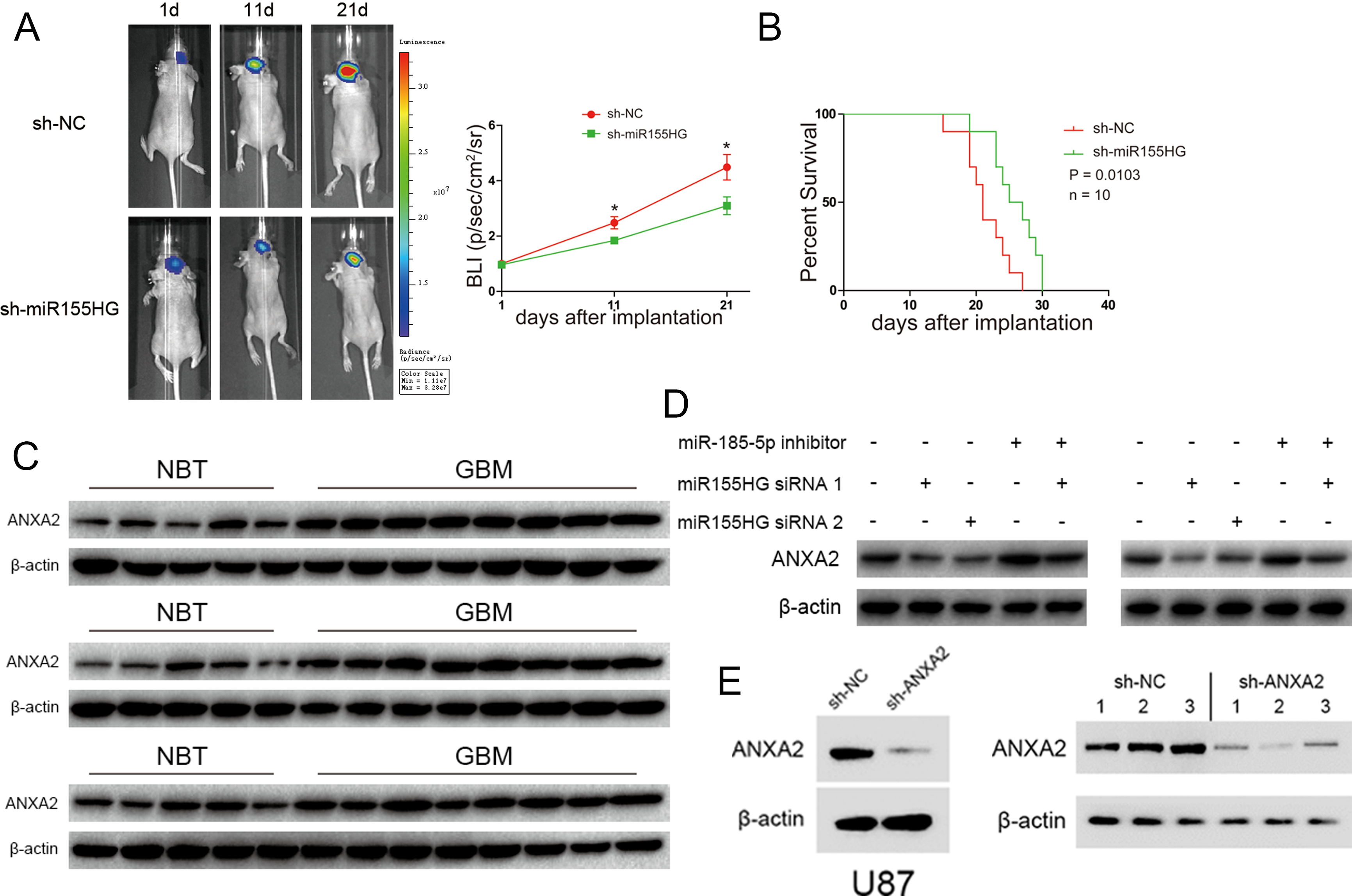

Supplement: Supplementary file 1 — Figure S1. (A) U87 cells pretreated with a lentivirus with sh-miR155HG or sh-NC and a lentivirus containing luciferase were implanted in the brains of nude mice, and tumor formation was assessed by bioluminescence imaging. The bioluminescent images were measured at days 1, 11 and 21 after implantation. (B) Overall survival from two groups of nude mice injected with U87 cells, transfected with sh-NC or sh-miR155HG lentivirus, was determined by Kaplan-Meier survival curves, and a log-rank test was used to assess the statistical significance of the differences. (C) Expression levels of ANXA2 in GBM tissues and adjacent normal brain tissues were analyzed by western blotting and normalized to β-catenin. (D) The protein expression levels of ANXA2 were analyzed by western blotting after 48 h transfection in U87 and GP1 cells with scramble, miR155HG siRNA 1, miR155HG siRNA 2, miR-NC or inhibitor, respectively. (E) The effect of sh-ANXA2 in U87 cell and tumor tissue of nude mice after implantation were analyzed by western blotting. (E) Downregulating ANXA2 contributed to the reduction of p-STAT3 level in GBM cells. (TIF 9482 kb) [file 13046_2019_1132_MOESM1_ESM.tif]

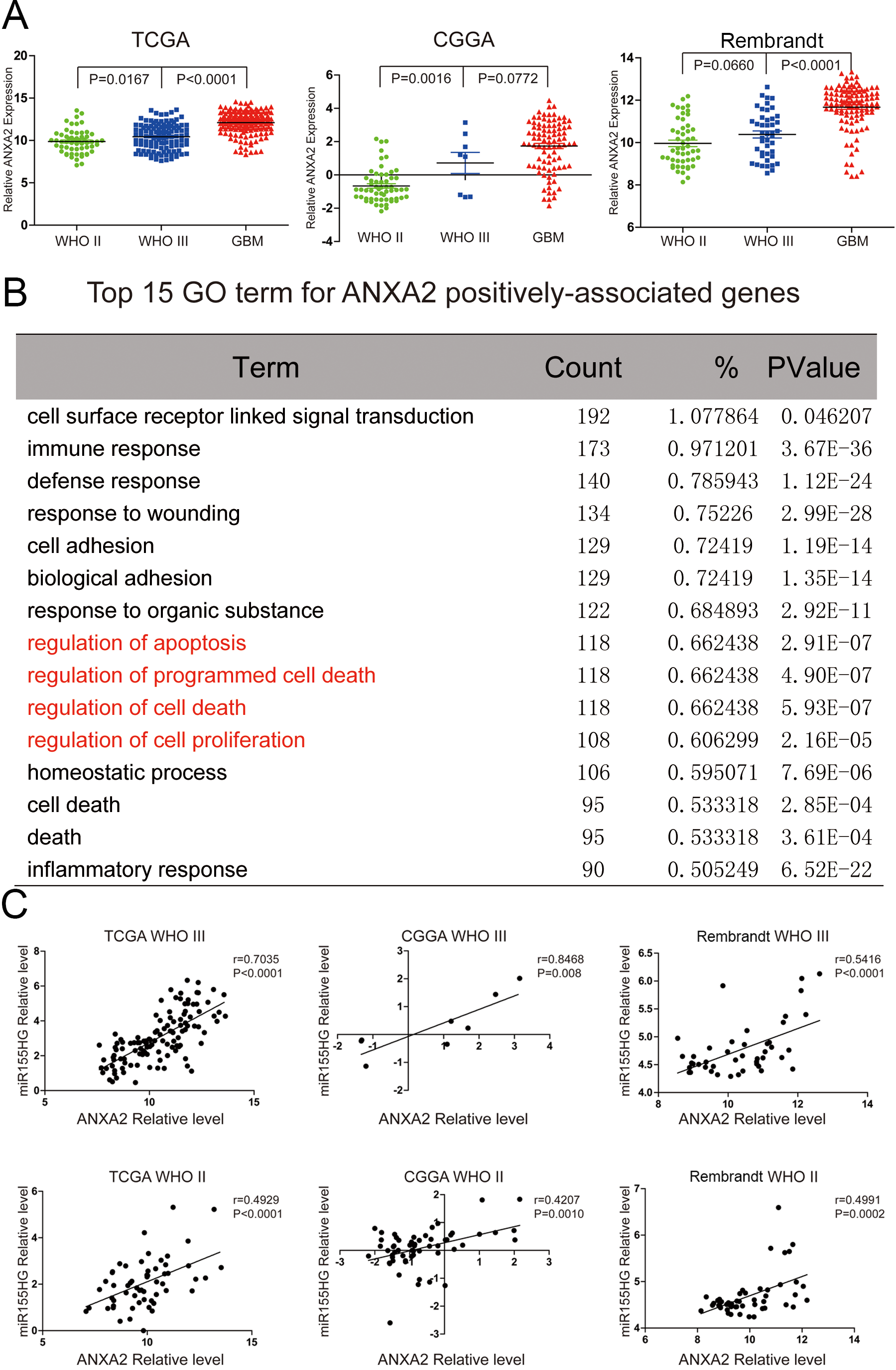

Supplement: Supplementary file 2 — Figure S2. (A) Expression of ANXA2 in TCGA, CGGA and Rembrandt astrocytoma database. (B) ANXA2 associated genes from overlapping CGGA, TCGA and Rembrandt databases were analyzed with gene oncology analysis. (C) ANXA2 positively correlates with miR155HG in WHOII/III astrocytoma specimens of three independent public database. (TIF 3895 kb) [file 13046_2019_1132_MOESM2_ESM.tif]

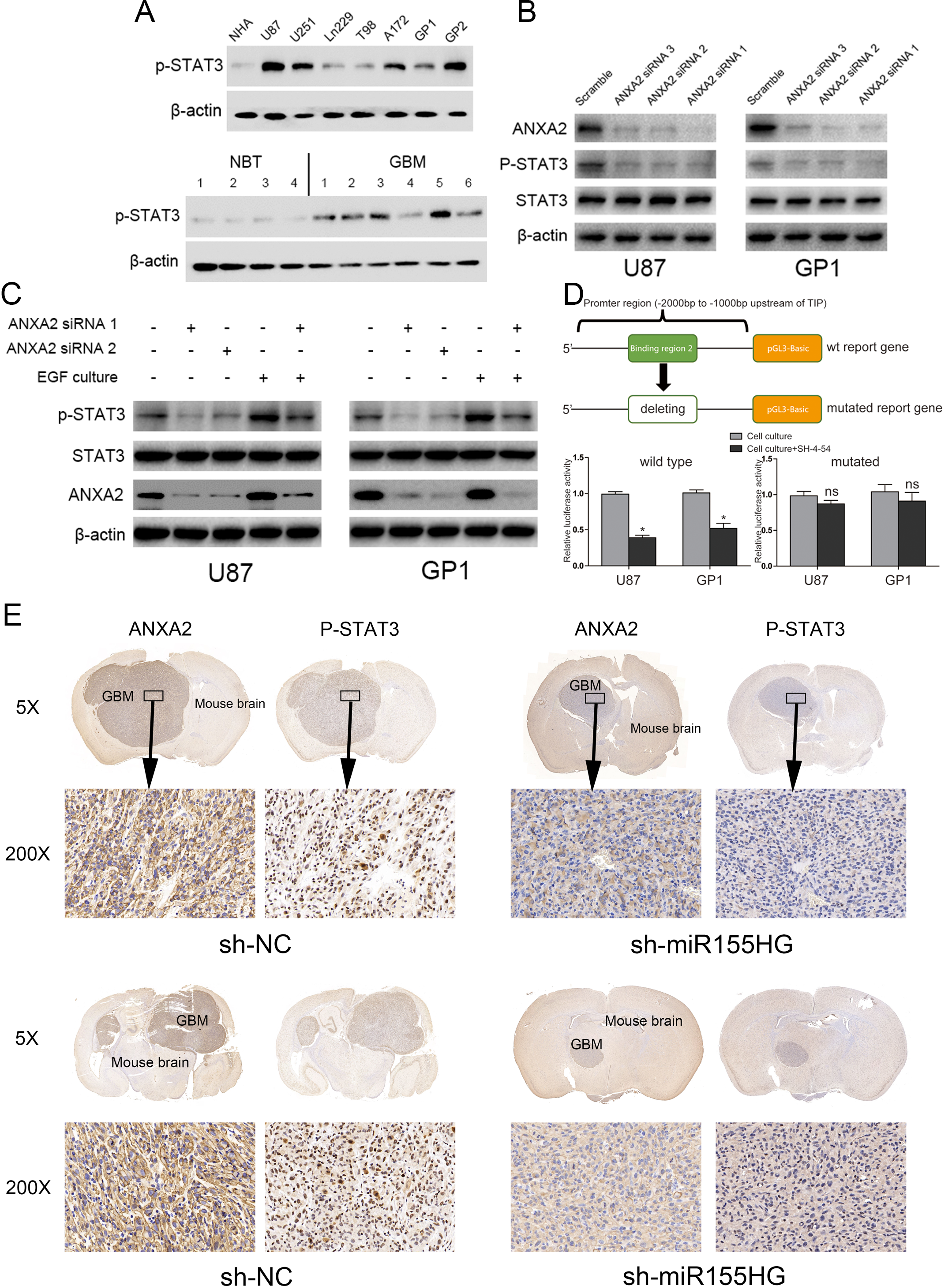

Supplement: Supplementary file 3 — Figure S3. (A) Expression levels of p-STAT3 in cell lines, GBM tissues and normal brain tissues were analyzed by western blot. (B) Downregulating ANXA2 contributed to the reduction of p-STAT3 level in GBM cells. (C) Overexpression STAT3 was constitutively activated by EGF in ANXA2-depleted GBM cells in U87 and GP1 cells. (D) Luciferase assays was performed after transfection with miR155HG promoter wt-pGL3 or miR155HG promoer mut-pGL3 as well as the internal control Renilla plasmid into U87 and GP1 cells. The cells then were treated with or without SH-4-54. Relative luciferase activity was analyzed after 48 h treatment. (*p < 0.05, **p < 0.01). (E) Two groups of representative immunohistochemical image of tumors from groups of nude mice implanted with U87 cells, transfected with a lentivirus with sh-miR155HG or sh-NC, were shown to compare the volume size of tumors and the expression of ANXA2 and p-STAT3 through serial slices of same section of tumor. (TIF 13681 kb) [file 13046_2019_1132_MOESM3_ESM.tif]
